# Supplementary material for: Disruption of the Unique ABCG-Family NBD:NBD Interface Impacts Both Drug Transport and ATP Hydrolysis
Source: Int J Mol Sci. 2020 Jan 23;21(3):759. doi: 10.3390/ijms21030759 (PMC7037313; doi:10.3390/ijms21030759)
Supplement: Supplementary file 1 [file ijms-21-00759-s001.pdf]

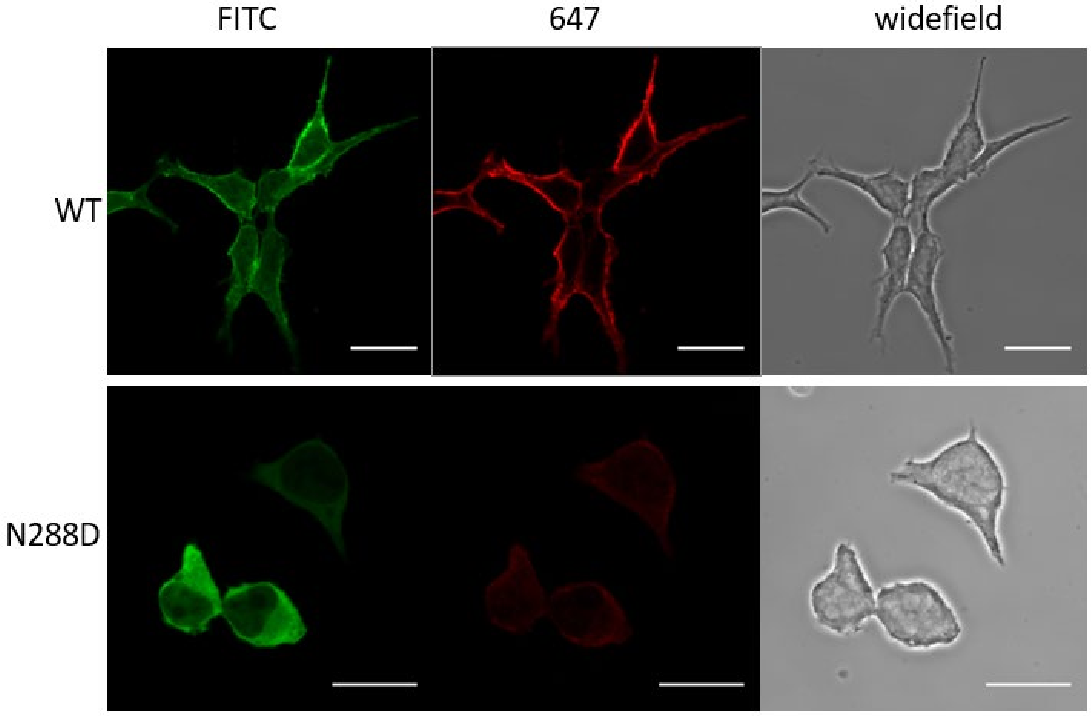

Supplementary Figure S1. Confirmation of intracellular localisation of N288D. HEK293T cells expressing WT (top) and N288D (bottom) ABCG2 isoforms were visualised for protein localisation by confocal microscopy in fixed cells (4% paraformaldehyde, 5 minutes) for both GFP expression (left hand panels) and for ABCG2 expression (middle panels, 5D3 antibody and AlexaFluor-647 coupled secondary antibody). Panels represent Visualisation of cells was conducted on an LSM710 confocal laser scanning microscope (Zeiss, Jena, Germany) equipped with a Plan-Apochromat 63×/1.40 Oil Ph3 DIC M27 objective and argon laser. For detection of sfGFP-ABCG2 isoforms, a 2% argon laser power was used with excitation set at 488 nm and emission collected between 500–530 nm (FITC channel). Scale bar is representative of 20 μm.
